# Long-term outcomes of endoscopic resection for duodenal neuroendocrine tumors

**DOI:** 10.1038/s41598-023-45243-8

**Published:** 2023-10-20

**Authors:** Kiyoun Yi, Gwang Ha Kim, Su Jin Kim, Cheol Woong Choi, Moon Won Lee, Bong Eun Lee, Geun Am Song

**Affiliations:** 1 Department of Internal Medicine, Pusan National University School of Medicine and Biomedical Research Institute, Pusan National University Hospital, 179 Gudeok-Ro, Seo-Gu, Busan, 49241 Korea; 2 Department of Internal Medicine, Pusan National University School of Medicine and Research Institute for Convergence of Biomedical Science and Technology, Pusan National University Yangsan Hospital, Yangsan, Korea

**Keywords:** Gastroenterology, Oncology

## Abstract

Duodenal neuroendocrine tumors (d-NETs) ≤ 10 mm in size, confined to the submucosal layer, without lymph node or distant metastasis, can be treated safely and effectively by endoscopic management. However, most results are based on limited data and short follow-up outcomes. Herein, we aimed to evaluate the short-term and long-term outcomes of endoscopic resection for d-NETs. We retrospectively analyzed 63 patients with 68 d-NETs who had undergone endoscopic resection at two hospitals between January 2009 and December 2021. *En-bloc* resection, endoscopically complete resection, and histopathologically complete resection rates were evaluated as short-term outcomes. Furthermore, long-term outcomes were analyzed in 46 patients with 50 d-NETs with a follow-up period of > 1 year. The overall *en-bloc*, endoscopically complete, and histopathologically complete resection rates were 92.6% (63/68), 100% (68/68), and 69.1% (47/68), respectively. Tumor size (> 5 mm) was the only predictive factor for histopathologically incomplete resection (*p* = 0.015). The procedure-related bleeding and perforation rates were 0% and 5.9%, respectively. No recurrences were observed in patients with histopathologically complete resection and those with histopathologically incomplete resection at a median follow-up period of 48 months (range 12–132 months). Endoscopic resection for d-NETs ≤ 10 mm in size, limited to the submucosal layer, and without lymph node or distant metastasis provides favorable long-term outcomes when endoscopically complete resection is achieved.

## Introduction

Neuroendocrine tumors (NETs), previously named carcinoid tumors, are rare tumors found most commonly in the gastrointestinal tract^1,2^. Within the gastrointestinal tract, most NETs are located in the small intestine, rectum, and stomach^3^. Duodenal NETs (d-NETs) have recently been increasingly recognized due to the widespread use of upper gastrointestinal endoscopy and thorough observation of the duodenum^4^. However, the management of d-NETs is not yet standardized because of their largely unknown natural history^5^. The European Neuroendocrine Tumor Society (ENETS) recently recommended that patients with d-NETs of periampullary location or size > 2 cm should undergo surgical resection^6^. ENETS also suggests that d-NETs ≤ 10 mm in size, confined to the submucosal layer, without lymph node or distant metastasis, can be treated safely and effectively using endoscopic management.

Commonly used endoscopic resection methods include endoscopic mucosal resection (EMR), EMR with a ligation device (EMR-L), EMR after circumferential precutting (EMR-P), and endoscopic submucosal dissection^7,8^. Due to the development of endoscopic techniques, there have been increasing reports on endoscopic resection of d-NETs ≤ 10 mm in size^1,9–12^. Accordingly, many studies have shown that EMR is an appropriate method for treating d-NETs ≤ 10 mm in size; however, most results are based on limited data and short-term outcomes. Therefore, the present study aimed to investigate the short- and long-term outcomes of endoscopic resection for d-NETs with a relatively large number of cases and long follow-up periods.

## Patients and methods

### Study population

**Figure 1:**
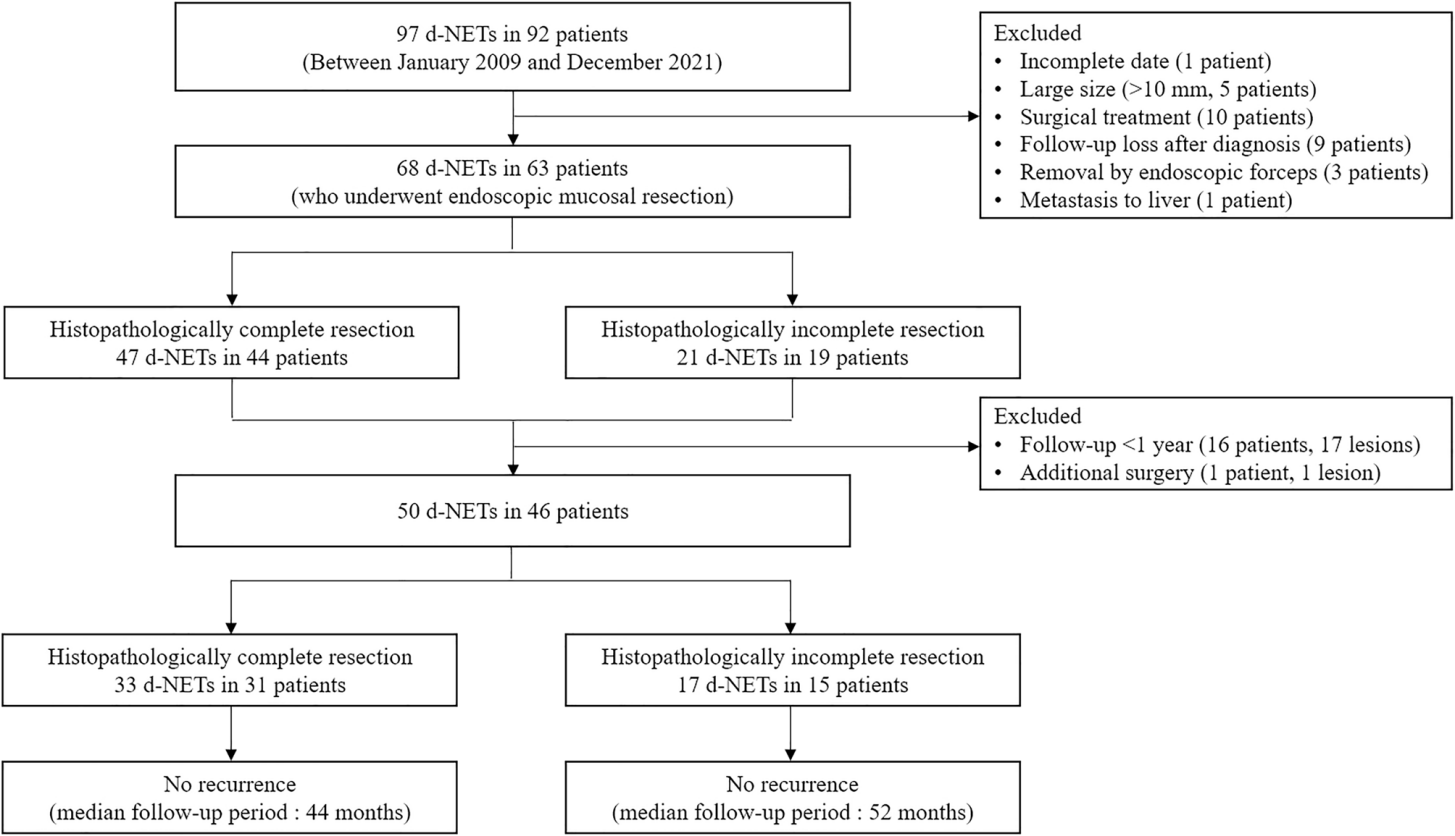
Flowchart showing patient inclusion in the study. *NETs* neuroendocrine tumors.

Between January 2009 and December 2021, 92 patients with 97 d-NETs were retrospectively enrolled at two tertiary hospitals (Pusan National University Hospital and Pusan National University Yangsan Hospital, Korea). The indications for endoscopic resection were d-NETs < 10 mm in size, limited to the submucosal layer on endoscopic ultrasonography using a 20-MHz catheter probe (UM3D-DP20-25R, Olympus, Tokyo, Japan), without lymph node or distant metastasis on abdominopelvic computed tomography (CT). Among the 92 patients, 29 were excluded due to incomplete data (*n* = 1), large size (> 10 mm, *n* = 5), surgical treatment (*n* = 10), follow-up loss after diagnosis (*n* = 9), removal by endoscopic biopsy forceps (*n* = 3), and metastasis to the liver at the time of diagnosis (*n* = 1). Consequently, 68 d-NETs in 63 patients treated with EMR, EMR-L, and EMR-P were analyzed in this study (Fig. 1). This study was conducted in accordance with the Declaration of Helsinki, and the study protocol was reviewed and approved by the Institutional Review Board (IRB) of the Pusan National University Hospital (IRB number: 2211-021-121). The requirement for acquisition of informed consent from patients was waived by the IRB owing to the retrospective nature of this study.

### Endoscopic resection

EMR, EMR-L, and EMR-P were performed with a conventional single-channel endoscope (GIF-Q260, GIF-H260, Olympus) while the patient underwent intravenous, conscious sedation (midazolam and/or meperidine) as stated previously^12^. For EMR, a solution comprising normal saline, epinephrine (0.025 mg/mL), and indigo carmine dye was first injected into the submucosal layer, and the lesion was then resected using a snare. In EMR-L, the lesion was aspirated into the ligation device (Stiegmann-Goff ClearVue, ConMed, Boston, MA, USA), followed by the deployment of a rubber band. Snare resection was performed below the rubber band using a mixed electrosurgical current. For EMR-P, a flex knife (Fixed flexible snare™, Kachu Technology, Seoul, Korea) was used to make markings 2 mm outside the tumor margin. After injecting a saline solution into the submucosal layer around the lesion, a circumferential incision was made using the flex knife. Then, snare resection was performed with an additional injection beneath the lesion.

### Histopathological evaluation

Formalin-fixed resected specimens were sectioned serially at 2-mm intervals and assessed for tumor involvement at the horizontal and vertical margins. In addition, the depth of invasion, histopathological grade, tumor size, and lymphovascular invasion were evaluated microscopically. Immunohistochemical staining with synaptophysin, chromogranin A, and CD56 was performed for all cases, and the neuroendocrine differentiation and mitotic rate of the tumor were evaluated. Ki-67 staining was also performed to evaluate the tumor cell proliferative activity. The tumors were classified as G1, G2, or G3 based on the mitotic rate and Ki-67 index. Diagnosis was performed in accordance with the guidelines valid at the time of the patient presentation and the diagnosis was re-evaluated for this study based on the available data according to the 2019 World Health Organization (WHO) classification^13^.

### Definition of en-bloc and endoscopically and histopathologically complete resections

The resections were defined as *en-bloc*, endoscopically complete, and histopathologically complete resection. *En-bloc* resection was defined when the lesion was resected as a single piece. Endoscopically complete resection was defined when residual tumors at the resection site could not be identified by endoscopy, regardless of whether *en-bloc* resection was performed or not. Histopathologically complete resection was defined when all of the following conditions were satisfied: (1) performed by *en-bloc* resection, (2) classified as a well-differentiated NET (G1, G2) according to the WHO classification, (3) limited to the submucosal layer, (4) absence of horizontal and vertical margin involvement, and (5) absence of lymphovascular invasion.

### Follow-up after endoscopic resection

The patients were followed-up with endoscopy, with or without biopsies and abdominopelvic CT, every 6 months for 2 years after endoscopic resection. Subsequently, annual endoscopy and abdominopelvic CT were performed to check the occurrence of local recurrence, lymph node metastasis, and distant metastasis.

### Statistical analysis

Variables are expressed as medians, ranges, and proportions. The differences in clinicopathologic characteristics between histopathologically complete and incomplete resections were assessed using χ^2^ or Fisher’s exact test. A *p*-value < 0.05 was considered statistically significant. Statistical calculations were performed using IBM SPSS version 27.0 for Windows (IBM Co., Armonk, NY, USA).

## Results

### Patient and duodenal neuroendocrine tumor characteristics

The baseline characteristics of 68 d-NETs in 63 patients who underwent endoscopic resection are listed in Table 1. Of all patients, 39 were male and 29 were female, and the age ranged from 33 to 82 years, with a median age of 59 years. Almost all tumors (57/68, 83.8%) were found in the bulb, and 11 (16.2%) were in the second portion of the duodenum. Macroscopically, 19 tumors were type I, 46 were type IIa, and 3 were type IIb, according to the Paris endoscopic classification^14^. Fifty-nine patients had a single tumor, 3 had 2 tumors, and 1 had 3 tumors. The median tumor size was 6 mm (range 2–10 mm). Histopathologically, 3 tumors were confined to the mucosa, and 65 extended to the submucosa. According to the WHO classification, 57 (83.8%) tumors were G1 and 11 (16.2%) were G2. EMR was performed in 22 tumors, EMR-L in 41, and EMR-P in 5, according to the endoscopist’s preference.

**Table 1:** Characteristics of 68 duodenal neuroendocrine tumors in 63 patients who underwent endoscopic resection.

Characteristics	
Sex, *n* (%)	
Male	39 (57.4)
Female	29 (42.6)
Median age (years, range)	59 (33–82)
Tumor location, *n* (%)	
Bulb	57 (83.8)
Second portion	11 (16.2)
Macroscopic shape^a^, *n* (%)	
I	19 (28.0)
IIa	46 (67.6)
IIb	3 (4.4)
Tumor number, *n* (%)	
Single	59 (93.7)
Two or more	4 (6.3)
Tumor size, *n* (%)	
< 5 mm	16 (23.5)
5–10 mm	52 (76.5)
Invasion depth, *n* (%)	
Mucosa	3 (4.4)
Submucosa	65 (95.6)
Histopathological grade^b^, *n* (%)	
G1	57 (83.8)
G2	11 (16.2)
Treatment methods, *n* (%)	
EMR	22 (32.4)
EMR-L	41 (60.3)
EMR-P	5 (7.3)

*EMR* endoscopic mucosal resection; *EMR-L* EMR with a ligation device; *EMR-P* EMR after circumferential precutting.

^a^According to the Paris endoscopic classification^12^.

^b^According to the 2019 World Health Organization classification^11^.

### Short-term outcomes of endoscopic resection

Overall short-term outcomes of endoscopic resection are listed in Table 2. The median procedure time was 6 min (range 1–30 min). *En-bloc* resection was achieved in 63 tumors (92.6%) and piecemeal resection in 5 (7.4%). Endoscopically complete resection was achieved in all tumors. However, the histopathologically complete resection rate was 69.1% (47/68) due to horizontal or vertical margin involvement in 19 cases and/or lymphovascular invasion in 4 cases. Intraprocedural perforation was noted in 4 cases (5.9%); 3 in EMR and 1 in EMR-L. All cases were successfully closed using hemoclips. Delayed adverse events such as delayed bleeding and stenosis did not occur in any case. Four patients with lymphovascular invasion were recommended additional surgery; however, only one underwent surgery. Three patients refused because of co-morbidities and advanced age.

**Table 2:** Short-term outcomes of endoscopic resection for duodenal neuroendocrine tumors.

Median procedure time (min, range)	6 (1–30)
*En-bloc* resection rate	92.6% (63/68)
Endoscopically complete resection rate	100% (68/68)
Histopathologically complete resection rate	69.1% (47/68)
Causes for histopathologically incomplete resection	
Horizontal/vertical/ both involvement	3/6/10
Lymphovascular invasion	4^a^
Adverse events, *n* (%)	
Bleeding	0 (0)
Perforation	4 (5.9)

^a^Involvement of tumors in the horizontal margin or vertical margin was observed in one lesion each.

### Factors associated with histopathologically incomplete resection

There was no significant difference in tumor location, macroscopic shape, invasion depth, histopathological grade, and treatment methods between histopathologically complete and incomplete resections (Table 3). However, histopathologically incomplete resection was higher in tumors with 5–10 mm than in those with < 5 mm (38.5% [20/52] vs. 6.3% [1/16], *p* = 0.015).

**Table 3:** Univariate analysis of predictive factors for histopathologically incomplete resection.

Variables	Histopathologically complete resection (*n* = 47)	Histopathologically incomplete resection (*n* = 21)	*p* value
Location			0.777
Bulb	39	18	
Second portion	8	3	
Macroscopic shape			0.494
I	13	6	
IIa	31	15	
IIb	3	0	
Tumor size			0.015
< 5 mm	15	1	
5–10 mm	32	20	
Invasion depth			0.925
Mucosa	2	1	
Submucosa	45	20	
Histopathological grade			0.319
G1	38	19	
G2	9	2	
Treatment methods			0.843
EMR	16	6	
EMR-L	28	13	
EMR-P	3	2	

*EMR* endoscopic mucosal resection; *EMR-L* EMR with a ligation device; *EMR-P* EMR after circumferential precutting.

### Long-term outcomes of endoscopic resection

Of the 63 patients with d-NETs, 16 patients with a follow-up period of < 1 year and 1 patient who underwent surgical treatment immediately after EMR were excluded from the analysis of long-term outcomes after endoscopic resection. Accordingly, 50 d-NETs in 46 patients were included in the analysis of the long-term outcomes after endoscopic resection (Fig. 1). During a median follow-up period of 48 months (range 12–132 months), neither local tumor recurrence nor metastasis occurred in any patient.

Among the 33 d-NETs in 31 patients with histopathologically complete resection, local recurrence or metastasis did not occur in any patient during a median follow-up period of 44 months (range 12–132 months). Among the 17 d-NETs in 15 patients with histopathologically incomplete resection (1 d-NET in 1 patient with horizontal margin involvement [a follow-up period of 23 months], 5 d-NETs in 5 patients with vertical margin involvement [a median follow-up period of 78 months; range 35–130 months], 8 d-NETs in 6 patients with horizontal and vertical margin involvement [a median follow-up period of 67.5 months; range 13–90 months], and 3 d-NETs in 3 patients with lymphovascular invasion [a median follow-up period of 38 months; range 18–48 months]), local recurrence or metastasis also did not occur in any patient during a median follow-up period of 52 months (range 13–130 months).

## Discussion

The present study included 46 patients with 50 d-NETs who underwent endoscopic resection to analyze long-term outcomes. Local recurrence or metastasis did not occur in any patient during a median follow-up period of 48 months. These results demonstrated favorable long-term outcomes for patients with d-NETs treated with endoscopic resection. Considering that d-NETs are rare tumors, the present study strongly supports the indication of endoscopic resection for d-NETs abovementioned in the ENETS guidelines^6^.

The possibility of metastasis is an essential factor to be considered when selecting endoscopic resection as the treatment modality for d-NETs^15^. Several studies have reported risk factors related to lymph node metastasis in d-NETs^16–18^; the main risk factors are tumor size > 10 mm, lymphovascular invasion, WHO grade G2, and invasion beyond the submucosa. Therefore, in the present study, patients with d-NETs ≤ 10 mm in size, limited to the submucosal layer, and without lymph node or distant metastasis in imaging studies were included in the indication of endoscopic resection.

In the present study, endoscopically complete resection was accomplished in all cases; however, the histopathologically complete resection rate was 69.1%, similar to the results of previous studies^11,12,19^. The lower histopathologically complete resection rate compared to the endoscopically complete resection rate could be explained by the difficulty in histopathologically analyzing the horizontal and vertical margins of the resected specimens owing to cauterization artifacts^20,21^. Despite the histopathologically incomplete resection state, the electrocauterization effect could destroy remnants of tumor cells in the resection margins with heat generation during endoscopic resection^19,22^. The present study investigated the factors associated with histopathologically incomplete resection; the tumor size was the only statistically significant predictive factor for histopathologically incomplete resection. When the tumor size was ≥ 5 mm, the histopathologically complete resection rate was only 61.5%. Although endoscopically complete resection seems sufficient to treat d-NETs in the present study, endoscopists should pay more attention to increasing the histopathologically complete resection rate during the procedure.

Intraprocedural perforation occurred in 4 cases, which were closed successfully by hemoclips. None of the patients experienced delayed adverse events such as delayed bleeding and stenosis. These results are consistent with previous results that endoscopic resection is a safe treatment modality for d-NETs^12,22^.

We investigated the long-term outcomes of patients who underwent endoscopic resection for d-NETs. We included a relatively large number of patients at two hospitals and analyzed the data of patients with follow-up periods longer than 1 year. No recurrence or metastasis occurred in 33 d-NETs in 31 patients with histopathologically complete resection during the median follow-up period of 44 months. Our results strongly agree with the current indication of endoscopic resection for D-NETs suggested in previous studies^12,22^.

The current ENETS guidelines recommend additional surgery for cases with resection margin involvement after endoscopic resection^6^; however, studies supporting these guidelines were insufficient due to the rarity of the d-NETs. Our policy for patients with histopathologically incomplete resection is follow-up with endoscopy and abdominopelvic CT every 6 months for 2 years after endoscopic resection and then annually, without additional surgery, when endoscopically complete resection is achieved, the invasion depth is limited to the submucosa, and lymphovascular invasion is absent. In the present study, we included 17 d-NETs in 15 patients with histopathologically incomplete resection who did not undergo additional surgery in the analysis of long-term outcomes. Neither local recurrence nor metastasis occurred in 1 d-NET in 1 patient with horizontal margin involvement, 5 d-NETs in 5 patients with vertical margin involvement, and 8 d-NETs in 6 patients with horizontal and vertical margin involvement during a median follow-up period of 23, 78, and 67.5 months, respectively. These results are consistent with the results of previous studies demonstrating that even in cases in which histopathologically incomplete resection was performed, if endoscopically complete resection was achieved, the invasion depth was limited to the submucosa, and lymphovascular invasion was absent, recurrence did not occur during the follow-up period^12,19,22,23^. Conversely, the situation is different in cases with lymphovascular invasion. We strongly recommended additional surgery to 4 patients with lymphovascular invasion after endoscopic resection, but 3 patients refused to undergo surgery due to co-morbidities and advanced age. Although lymph node and distant metastases did not still occur in these patients during a median follow-up period of 38 months, similarly to the results of a previous study^22^, regular and continuous long-term follow-up is mandatory.

This study has several limitations. First, this was a retrospective study that evaluated the short-term and long-term outcomes of endoscopic resection for d-NETs. Therefore, a bias occurred when selecting patients and retrospectively reviewing the outcomes. Second, the patients with d-NETs were selected for endoscopic resection according to the clinical opinions and patient needs at each hospital. Finally, although this study included a relatively large number of d-NETs and a long follow-up period to analyze endoscopic resection outcomes, the number of G2 lesions was small and d-NETs with a larger size were not included. Further studies are needed to investigate evidence-based, standardized guidelines for treating G2 lesions and intermediate-sized d-NETs (10–20 mm) because the optimal standard for the WHO grade and size in which endoscopic resection is feasible and safe is still controversial^24^.

In conclusion, endoscopic resection for d-NETs ≤ 10 mm in size, limited to the submucosal layer, and without lymph node or distant metastasis provided favorable short- and long-term outcomes when endoscopically complete resection was achieved. Further multicenter studies, including more patients with longer follow-up periods, are needed to confirm our results.

## Data Availability

The datasets used and/or analyzed during the current study are available from the corresponding author on reasonable request.
